# Evaluation of STEM students' spatial abilities based on a novel net cube imagination test

**DOI:** 10.1038/s41598-023-44371-5

**Published:** 2023-10-12

**Authors:** Anita Pawlak-Jakubowska, Ewa Terczyńska

**Affiliations:** 1 Faculty of Civil Engineering, Silesian University of Technology, 44-100 Gliwice, Poland; 2 Faculty of Architecture, Civil Engineering and Applied Arts, The University of Technology in Katowice, 40-555 Katowice, Poland

**Keywords:** Engineering, Civil engineering

## Abstract

This study aimed to determine the level of spatial ability among STEM students. A universal multiple-choice test was prepared. The validity of the test and the effectiveness of its application were tested. The test is an extension of those currently in use. It contains tasks on spatial perception, spatial visualization, mental folding, rotation of spatial elements, and representation of spatial elements on a plane. The test consists of 16 tasks showing a cube with lines located on the walls. The student's task was to determine the development of the cube and mentally construct a cube based on the development. The results of the test determined the level of progress of the group (105 participants), and showed that a significant number of students have difficulties in perceiving and working with a three-dimensional object. On average 55% of the questions were answered correctly. For the group tested, reading a flat drawing and determining axonometry proved easier than other task. Students who attended technical high school or had design experience scored better. During the course, measures to improve teaching were introduced. Spatial model work was strengthened and initial tasks were adjusted according to the level of the group. Emphasis on teamwork and consultation was introduced for those with the lowest scores. The applied modifications in classroom management had a good effect. The average of the final grade was B. The test is a useful tool for academics and students to study spatial ability and improve teaching activities for STEM students.

## Introduction

One of the most important qualities a student of engineering sciences should have is a well-developed perception of three-dimensional space. Observing both engineered objects and skillfully manipulating them in one’s imagination are important aspects of this skill. Spatial abilities allow student to solve engineering problems in the area of design, technical, technological, and mechanical issues during both their studies and future careers. Spatial imagination and spatial perception are the main factors, which significantly affect the ease of studying science subjects. Perceptiveness, logical thinking, the ability to mentally rotate and transform objects characterize people aspiring to work in the engineering profession. These skills are essential for making accurate decisions in the process of innovative solution of technical issues at the design stage. They are closely related to innate spatial abilities as well as spatial skills acquired in childhood and during education (especially in its early stages). However, experience shows that students of technical sciences not infrequently have problems with the correct perception of the world around them. The teacher, whose main task should be to skillfully impart knowledge and encourage work on the development of spatial imagination, has a large role to play in correcting these problems.

Learning outcomes to be achieved as part of the first year university engineering course in drafting geometry and engineering graphics include the development of spatial imagination and the correct perception of engineering objects. This requirement appears in most curricula for subjects related to geometry and engineering graphics at technical universities.

Great Greek philosophers like Plato and Aristotle dealt with space perception, spatial imagination, and human cognitive abilities. Creative thinking requires, among other things, the ability to mentally rotate simple two- or three-dimensional figures and the ability to manipulate complex objects. The forerunner of intelligence research was British anthropologist Francis Galton originally conceived the idea of spatial ability^1^. He was also the initiator of test research for analyzing human mental abilities. The introduction of graphic tests undoubtedly supports the development of spatial imagination because both searching one’s imagination for a solution to a problem and subjecting potential solutions to analysis stimulates thinking and encourages finding the right answer.

Solving engineering issues often requires an interdisciplinary approach combining experience and knowledge from different fields as well as the skillful use of modern computer techniques. Nowadays, many computer software programs are capable of 3D modeling along with generation of animations and virtual walkthroughs, which also contribute to the promotion of spatial perception. However, several studies demonstrated that solid modeling alone is less effective for the development of spatial thinking^2,3^. Results from solid modeling were compared with traditional techniques—sketching, freehand drawing, or analysis of a spatial element illustrated on a flat sheet of paper supported, e.g., by a physical model—which gave significantly better results.

High quality education in science, technology, engineering, and mathematics (STEM) fields significantly affects the use of acquired knowledge and the ability to apply it in the workplace. Research in mathematics education in STEM fields is of great importance and is the foundation for all engineering courses. According to considerations by Tosto et al., “spatial ability predicts performance in mathematics and eventual expertise in science, technology and engineering”^4^ and “performance of spatial ability tasks correlates with mathematical task performance”^4^. A study^5^ “showed that the close association between spatial and mathematical abilities exists in childhood and adolescence”.

The connection between spatial ability and educational results received in engineering fields is examined in many studies^6–11^. The authors of the paper^7^ report that because of the significant impact on students' academic performance, understanding the cognitive processes behind the development and improvement of spatial abilities is an essential area of research for all STEM pedagogy.

The integration of spatial ability with engineering educational practices is presented in the research^12^. In addition, the correlation between spatial ability and engineering education has been discussed in the papers by^13–16^ and the importance of spatial ability in STEM education in the considerations by^10^. Researchers agree that it is important to mainly focus educational efforts on supporting and helping students develop their spatial abilities early in their studies and "spatial training should focus on improving students’ spatial ability"^17^. Spatial abilities improve through training^18^, therefore it is necessary to encourage students to use additional tools to support the education process of a future engineer like tests, physical or digital models.

An example of this is a study in mathematics education by^19^, which aimed to "foster students’ spatial skills and comprehension of applicational interconnections between architecture and mathematics, using geometry as a shared common language, and proposing paper models as tools for understanding the geometric construction of architectural shapes." The authors of the paper^19^ point out "the use of physical models as interactive teaching media to enhance geometric spatial knowledge." While a learning strategy using 3D printed models was presented in the paper^20^, where "the models supported students’ ability to connect theory to practice, mentally visualize engineering concepts, and develop engineering judgment. "

Therefore, spatial abilities play a very important role in obtaining positive academic results. In order to support “how to foster the development of spatial thinking skills, it is important to identify the fundamental differences between individuals who perform well and poorly on tests of spatial abilities and skills”^21^.

A review of the literature, structured definitions, and conducted research describing the evolution of spatial capabilities is presented in the study^10^.

Investigation of spatial imagination, spatial perception, and topics related to human intelligence are also presented in the work of^22^, in which spatial tasks were included in intelligence tests. Spatial abilities have been identified as a weighty element in other intelligence tests^23,24^. One study conducted by Quaiser-Pohl described and compared two tests used to assess spatial ability: the Mental Cutting test "Schnitte" and the Picture Rotation Test (PRT)^25^. The author of the theory of Multiple Intelligences, Howard Gardner, revolutionized the way we think about intelligence and learning^26^. People who can think pictorially are characterized by visual-spatial intelligence. They are able to use maps, tables, diagrams, are sensitive to detail, draw a lot, and present their ideas graphically. They skillfully use the ability to create images, spatial relationships, and visualizations in the mind. They orient themselves in three-dimensional space without any trouble. The topic of perception and spatial intelligence was also investigated in a paper^27^, which determines the relationship between cognitive styles and visual spatial intelligence. The author determined that "overall performance of the students at answering the questions indicates that a significant portion experienced difficulties in perceiving and working with three-dimensional geometrical objects. It was observed that some student had problems with depth perception and struggled with assigning a third dimension to planar figures"^27^.

Researchers like^28,29^ emphasize the division found in psychological research between spatial ability and spatial skills. They state that spatial ability can be understood as a talent, something a person is born with and possess without any training or exercise. On the other hand, spatial skills can be placed in the group of skills that can be trained, developed, practiced. In an article^30^ introduces the concept of spatial visualization skills and proposes their division into categories:

- spatial perception;
- spatial visualization;
- mental rotations;
- spatial orientation.

The relationships that occur in the study of spatial ability when an object is analyzed from different viewing angles or when it is in motion have also been studied^31^. On the basis of that analysis, it was determined that the mental skills to make a projective record of a spatial object created in the imagination are important. Also important is the ability to reproduce the shape and spatial form recorded in projections, such as rectangular ones, and also to combine individual elements into a whole.

Researchers and teaching staff at technical universities involved in the teaching of descriptive geometry, as well as subjects such as engineering geometry, engineering graphics, technical drawing, or geometric CAD design, extensively research the issues of spatial imagination development and research methods to verify this development. All this effort is aimed at striving to effectively prepare the student for the engineering profession.

At technical universities in subjects related to descriptive geometry, two tests are mainly used. The Mental Rotation Test (MRT) measures perceptual ability^14,32,33^ and the Mental Cutting Test (MCT) measures spatial imagination^14,34–36^. Other tests are also used for educational purposes that check the validity of practical thinking:

- Differential Aptitude Test: Space Relations (DAT:SR)^14,37^;
- Purdue Spatial Visualization Test (PSVT)^14,38–45^ which consists of three parts:
  - Development (PSVT-D);
  - Rotations (PSVT-R)^46^;
  - Views (PSVT-V);
- 3-Dimensional Cube (3DC)^14,47^;
- Mental Cutting Test “Schnitte”^15,48,49^;
- and Santa Barbara Solids Test, “online spatial test that assesses the ability to identify the two-dimensional cross section of a three-dimensional geometric solid, a skill that is important in many STEM disciplines”^50^.

Research into the perception of space and its impact on human development is complex and important in an era of change in the world around us. The situation today shows that the knowledge of descriptive geometry is even more necessary than before^51^.

Research fundamentals were the works in which the authors used a similar technique—a multiple-choice test with tasks (of spatial ability issues) in which only one answer is correct. In one investigation^52^, the authors studied the effects of a course in drafting geometry on spatial vision skills among first-year students of the Faculty of Civil Engineering at the Košice University of Technology. The same test was used before and after the geometry course. Students were required to mentally fold two-dimensional (2D) patterns and choose the correct 3D objects. The task was chosen from a prior study^23^. The test was multiple-choice in which only one answer was correct. Similarly, in the paper^43^, the same system was used to test the students' level of perception of space. To measure this ability, the PSVT -D was used, where spatial model developments are employed in the questions. The PSVT was also used in another study^44^, in which the authors find support for the claim that studying computer graphics has an impact on visual-spatial abilities, making them stronger for most of the students. DAT:SR is a test in which the questions introduce a solid net (development), on the basis of which test takers must build a spatial element in their imaginations. The test was used by the authors of the paper^53^, who wanted to measure of the level of elaborate spatial imagination in first-year mechanical engineering students. The work of^14^ also fits into this area of research. Another research paper is an article^54^ that examined how levels of spatial ability impacted both performance and approaches to problem solving.

The considerations undertaken in the work represent research related to the determination of spatial abilities related to spatial perception, spatial visualization, mental folding and rotation among students. The issues is timely and are related to all engineering and technical sciences and the topics fall within the area related to the applications of applied geometry and graphics, geometry and graphics education and teaching methodology. The results of the research serves to improve and strengthen science education.

In higher education, including technical studies, education methods have undergone rapid change. These shifts are related to technological and generational development. Technological development has resulted in a generation of young engineers who from an early age use images seen on phones, tablets, and computers. The changes force adaptation in the conduct of teaching activities and motivate the search for both more flexible methods of imparting knowledge and for ways to test the level of spatial ability of students before they begin their studies. The task of the university teacher is to follow trends and adapt to them, which includes preparation of educational material that will allow students to understand the topics covered.

The aim of the present research was to develop a universal tool for checking (testing) students acquired skills in spatial ability and the correctness of practical thinking after completing the first part of a course of drafting geometry in architectural design. This course is taught in the first year of study and lasts two semesters. During the first semester students learn about mapping methods, Mongean projection, and method representation of point, line, and plane with auxiliary views application. They also learn projection methods used in technical practice in order to represent three-dimensional objects on a two-dimensional plane (e.g. orthographic and parallel projection, perpendicularity, measuring distances and dihedral angles). Other topics include the construction of plane polygons, common or perpendicular elements, along with the application of the trapezoidal layout of the projective plane or the construction of rotation of the plane by any angle. In the second semester, issues related to polyhedra and their properties (prism, pyramid, roof skeletons and developments) and surfaces of revolutions (sphere, cylinder, cone) are discussed. The course begins with the administration of a classical test (MCT), which is designed to measure the level of spatial imagination of students before teaching begins.

### Need for research

In our opinion, among the available tests (MRT; DAT:SR; PSVT-D, R, V; 3DC or The Mental Cutting Test "Schnitte") there was a missing element for testing students' preparation for the start of the second semester and reflected several simultaneous needs. An important skill was the combination of the ability to rotate a spatial element (e.g., a cube) in one's mind, with simultaneous analysis of the lines occurring on the individual faces of the element and also determining their views, as well as decomposing the spatial element into a plane form (i.e., determining the development of the spatial element). The aforementioned tests have the possibility to separately check abilities such as development (PSVT-D; DAT:SR), rotations (MRT; PSVT-R; 3DC) or views (PSVT-V); however, this requires the use of several tests separately. With the needs in mind, a search was undertaken to construct a test that would bring together all the aspects mentioned above and also support the processes of learning, teaching, and development of students and teachers. Thus, the test can become a helpful tool for future recipients and relevant in the process of engineering education. We believe that research in the area of the discussed issue is important due to the need to create a universal test as an alternative to those currently used (MRT; DAT:SR; PSVT-D, R, V; 3DC or The Mental Cutting Test "Schnitte").

It is hypothesized that a universal test to check the spatial ability of the student in terms of rotating a spatial element in thought, correctly determining views, and creating developments is a useful tool for teaching of a course in descriptive geometry and could also aid in the student's educational activities.

The goals of the research (RG) were as follows:

- (RG1) develop a universal multiple-choice test that tests the student's level of proficiency in the rotation of a spatial element in thought, the correct determination of views, and the creation of expansions of a spatial element.
- (RG2) obtain information on how to conduct further classes (semester II of the first-degree program) in the subject of drafting geometry in architectural design for the surveyed group of students and prepare them for further work;
- (RG3) answer questions about the differences among students in spatial visualization according to age, gender, and design experience.

The following research questions (RQ) are related to the objectives:

- (RQ1) What percentage of test takers will give the correct answer? On this basis, it is possible to indicate the number of people who will require, for example, additional consultations or whether a given group should participate in workshops supplementing knowledge on given issues.
- (RQ2) Is there a difference between students graduating from junior high schools and technical secondary schools in terms of the test results obtained?
- (RQ3) Is there a difference in the test results due to a gender?
- (RQ4) Is there a difference in the test results between students with and without work experience?

The main research problems in the work were checking the validity of the test and its tasks, and also determining the test’s potential in terms of usefulness for among first-year students of technical universities. Indirect research problems in the work were implementing the tasks contained in the test, determining the degrees of difficulty for the tasks, and testing tasks on the target group of students.

## Methods

The study uses a research technique in the form of a self-developed test (Appendix 1), which is an extension of those currently used (MRT; DAT:SR; PSVT-D, R, V; 3DC or the Mental Cutting Test "Schnitte"). The test consists of tasks concerning the perception of space, visualization of space, mental folding and rotation of a spatial element, and mapping a spatial element onto a plane. Based on it, an experimental (heuristic) study was conducted to determine the ability of perception and also the correctness of practical thinking. The obtained data from the conducted test were used to elaborate the results in the form of graphs and tables. The test includes tasks in graphical form made using CAD computer software.

### Participants

The research was conducted at the Katowice University of Technology in 2022 on a study group of 105 participants (Table 1). The participants were full-time and part-time second semester students of the first degree program in architecture. The course dealt with topics from the subject of descriptive geometry in architectural design. As in^55^, the study group of students was divided by age, gender^56^, and type of high school completed (dividing into high school and technical school), or by design experience or lack thereof. The study was carried out in one sample, after the course of Descriptive Geometry in Architectural Design was completed during the first semester and before the beginning of its second part in the second semester of the study.

**Table 1:** Test participants—number of students involved in the course.

	Number of participants	Age of participants				
		19–25	26–30	31–35	36–40	Over 40
Female	64	50	6	4	2	2
Male	41	31	6	2	2	0

Among the study participants, 60.95% were women and 39.05% were men. The largest age cohort, 81 surveyed, were between the ages of 19 and 25. This age group predominated because most students took up studies immediately after finishing their high school education. The study group included 45 people who had completed an apprenticeship at a technical school, or were employed in project offices. Of the participants, as many as 74 students graduated from high school.

### Material

On the basis of literature research, available tests (MCT; MRT; DAT:SR; PSVT-D, R, V; 3DC; The Mental Cutting Test "Schnitte"), and many years of teaching experience in teaching courses in drawing geometry and engineering graphics, a test was developed that checks spatial abilities and also the correctness of practical thinking.

### Test

**Figure 1:**
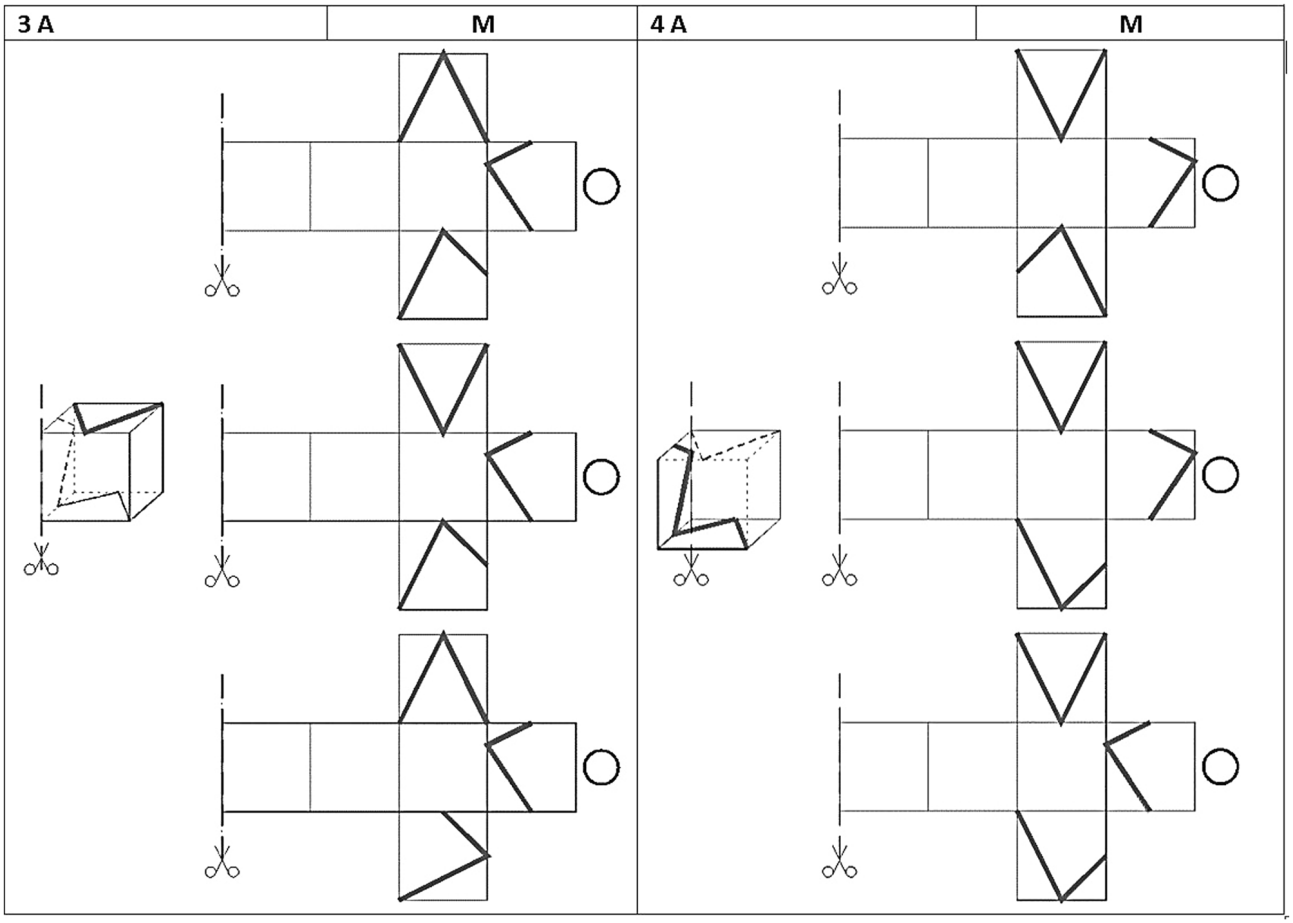
Depending on the cut line introduced, the student determines the development of the cube walls on the basis of a cubic element.

Based on experience with existing tests, a cube was chosen as the simplest spatial form for the composition of tasks as a spatial element. Lines were introduced on the individual walls of the cube and positioned in a variety of ways. The student's task was to imagine the development of the cube (Fig. 1). In order to determine the location of the lines on the individual walls, the student must skillfully use the individual views of the cube (mentally perceive different faces of the cube and determining the location of the lines) and also make mental rotations of the individual walls with the lines until a flat net is obtained. The test developed is multiple-choice and consists of 16 questions. Each question gives 3 answers, only one of which is correct.

**Figure 2:**
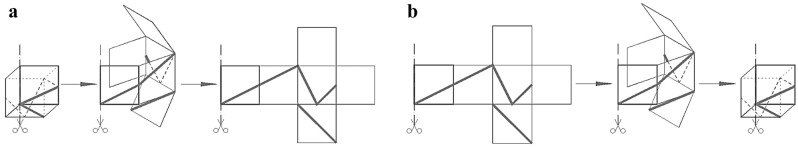
Two parts of the test. (**a**) Part A- the way the net is unfolded. (**b**) Part B—the way the net is folded.

The introduction of cutting lines and the definition of the direction and method of unfolding both give clear information on how to proceed in order to obtain a flat net from a cubic form (Fig. 2a). The eight tasks in which the student must decompose the spatial element into a flat net are found in the first part of the test, labeled A. The second part of the test consists of another eight tasks, marked part B, which ask for the inverse situation. Based on the flat net, the student must transform it into a cube with lines drawn on the walls (Fig. 2b).

The test contains a pool of questions of varying difficulty, about which the student is not informed. The difficulty of the questions is twofold.

**Figure 3:**
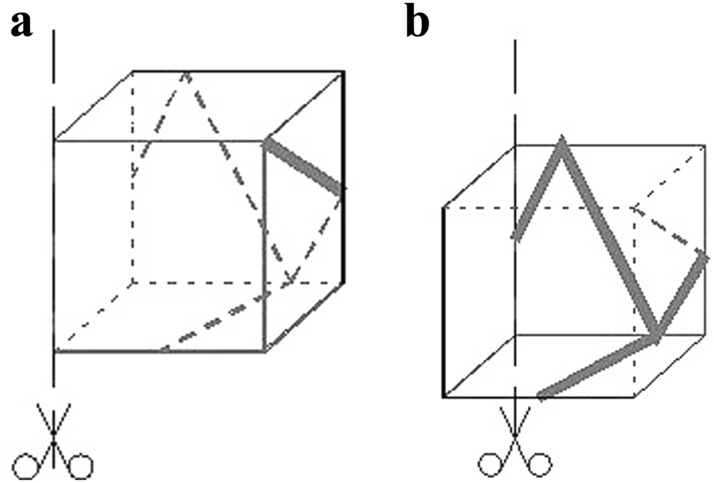
Two ways of constructing a task. (**a**) The observer of the cube element looks at the cube in which the front wall is a front view. (**b**) The same element but the front view is represented by its back wall.

- The first difficulty relates to the view and different ways of perceiving the front wall assuming that the observer does not change his position (Fig. 3). The inserted cut line determines how the element is observed and determines the front wall of the spatial element. Usually the easier image for students to analyze is the one in which the axonometry of the element is shown as in Fig. 3a. In contrast, the case presented in Fig. 3b is less intuitive and usually more difficult to execute and analyze. Thus, in Part A, the questions identified as easy are 1A, 3A, 5A and 7A with, in turn, 2A, 4A, 6A and 8A as difficult. In the second part B, the situation is similar, however, the student determines the spatial element based on the net. Here the easy questions are 9B, 11B, 13B, 15B and the difficult ones 10B, 12B, 14B, 16B.
- The second difficulty concerns the introduced lines on the walls of the cube. Here too, two levels of difficulty were distinguished. Part A consisted of easy questions 1–4 (section AI) in which lines were introduced on three walls of the cube, and difficult questions 5–8 (section AII) in which lines were introduced on all walls of the cube. Part B consisted of section BI with easy questions 9–12, where lines were introduced on three walls of the cube, and section BII with difficult questions 13–16, where lines appear on all walls of the cube.

All tasks are allocated 20 min, of which 4 min are used to explain the idea of the test and how to solve it. The test is completed independently by the student and does not affect the grade for the subject.

### Physical model

After completing the test and determining the points earned, students have access to the tests and are encouraged to participate in consultations where they can make a physical model of the spatial element to better understand the example. For all assignments, the letter M is given next to the number as the model designation. Students can support themselves with the physical model of M by realizing it in the form of a grid, which can be folded into a cube. The student can apply lines to the walls of the cube according to the given case and observe the correctness of the solution. The grid (physical model M) is designed to encourage the student to analyze each case (even the most difficult ones) and solve it through independent deduction during the consultation.

### Reliability of the test

The reliability of the new test was validated in the 2019 course of descriptive geometry in architectural design. The research group was constituted by 57 students at the first semester of the architecture course. The test–retest method was used to determine the reliability of the research tools.

The test–retest reliability method is used by researchers^57^. It involves testing the same group of people two times using the same test. The second measurement should be performed after a certain time interval, e.g. a week, a month. The time interval is determined by the type of property being tested, which must not change significantly between measurements. Too long interval may result in the acquisition of new knowledge that falls within the scope of the research—this may have a significant impact on the results obtained during the second measurement.

A measure of reliability is the magnitude of the correlation coefficient between the two sets of results obtained from the two tests. A high correlation between the results indicates higher reliability of the test under study. Pearson correlation coefficient (PCC) *r* was determined according to the Eq. (1).

1 $${r}_{xy}=\frac{{\sum }_{i=1}^{n}\left({x}_{i}-\overline{x }\right)\left({y}_{i}-\overline{y }\right)}{\sqrt{{\sum }_{i=1}^{n}{\left({x}_{i}-\overline{x }\right)}^{2}}\sqrt{{\sum }_{i=1}^{n}{\left({y}_{i}-\overline{y }\right)}^{2}}}$$

where: *n*—is sample size, *x*_*i*_, *y*_*i*_—samples of variable x, y indexed with *i*.

The research was conducted in two stages. The first stage took place at the beginning of the first classes in a research group of 57 students. The second stage took place one week later at the beginning of the second classes in a research group of 52 students. The difference of one week between the surveys was dictated by the fact that the first class is an introductory class, where students receive general information about the subject. In turn, the second and subsequent classes deal with specific issues in the course that could affect the test results. Therefore, the test was conducted with an interval of one week.

**Figure 4:**
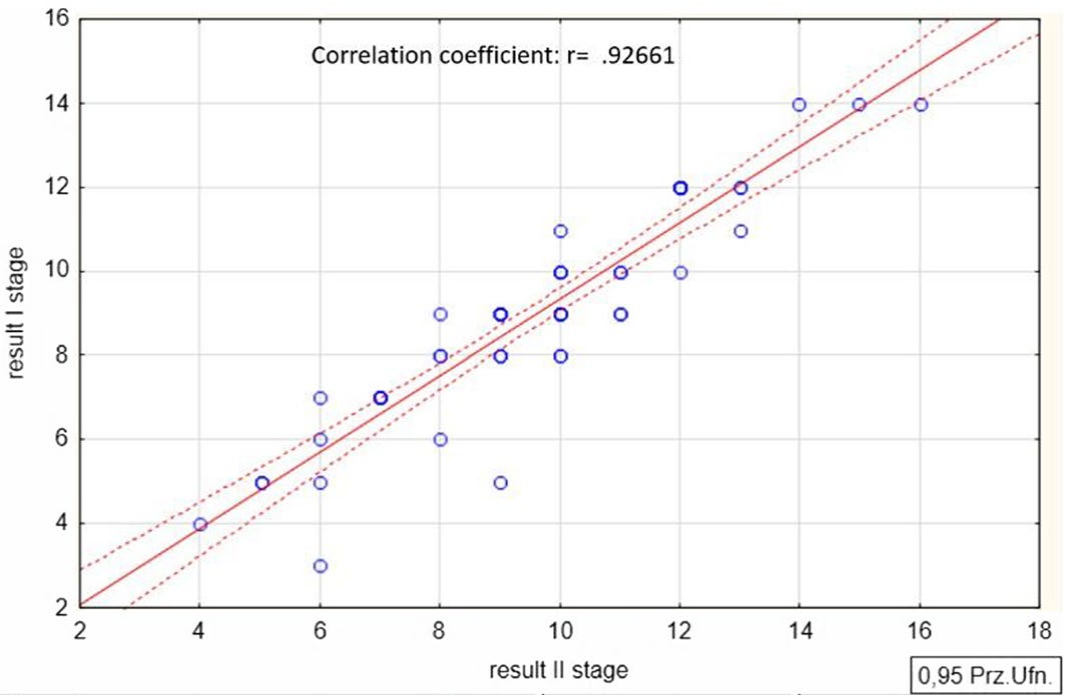
Scatter diagram made using Statistica 13.3 software for the results obtained from the test, conducted on two dates.

To determine the magnitude of the correlation coefficient *r*, the results for a research group *n* of 52 taking part in two stages were used. The magnitude of the correlation coefficient for the test results obtained in the two samples was 0.9266 (Table 2) and the scatter diagram is shown in Fig. 4.

**Table 2:** Correlation coefficient determined using Statistica 13.3 software.

	Number of participants (n)	Average	Standard deviation	PCC
I steage	52	8.9038	2.6066	r = 0.9266
II steage	52	9.5384	2.6602

The obtained values of the correlation coefficient demonstrate a very good reliability of the proposed test.

### Statistical analysis

To verify the randomness of the results of the collected data, statistical analyzes were performed. The research hypotheses relating to research questions RQ2, RQ3, RQ4 were verified. Analyzes were performed using TIBCO Statistica 13.3 software (TIBCO software Statistica 13.3). In statistical tests, measurements were made using the Shapiro–Wilk test (SW) to determine for the variables the normality of the distribution^58^. Statistical significance was determined using the Mann–Whitney U-test (MWU), which is a non-parametric test. In the analyses, the level of statistical significance was p < 0.05.

### Ethical statements

The ethical approval statements (Appendix 2):

- All methods were carried out in accordance with relevant guidelines and regulations, as well as maintaining procedures for anonymity and respecting human rights.
- Informed consent was obtained from all participants taking part in the study, who gave their consent to the test to check their spatial aptitude.
- The Institutional Ethical Committee of The University of Technology in Katowice approved all the experimental protocols followed in the study.

## Results

The main intention of giving the novel net cube imagination test to the students after the first part of the course was to verify the acquired spatial abilities and also to check the correctness of practical thinking. It turned out to be important to determine the level of preparation of students to start studying the second part of the course.

1. The first evaluation criterion concerned the difficulty of the tasks (Fig. 5):
  in section AI, the easiest task was task 1 (78.09% of correct answers), while the most difficult was task 4 (39.05% of correct answers);
  in section AII, the easiest task was task 5 (59.05% of correct answers), and the most difficult was task 6 (33.33% of correct answers);
  in section BI, the easiest task was task 9 (82.86% of correct answers), while the most difficult was task 12 (37.14% of correct answers);
  in the BII section, the easiest task was task 15—(71.42% of correct answers), and the most difficult was task 14 (51.42% of correct answers).

**Figure 5:**
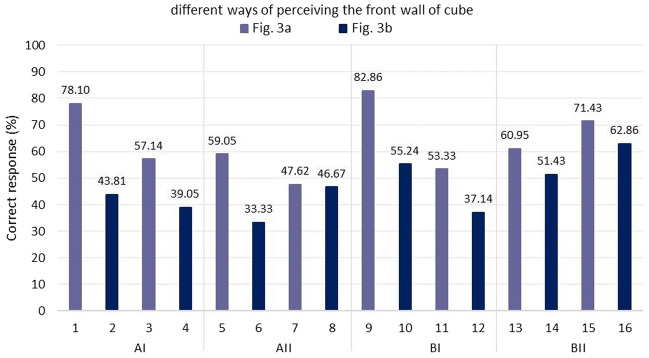
Percentage of people giving correct answers depending on the task number.
2. The second criterion was to determine the effect of which cube wall (front or back) is in the front view. The way of looking at the individual walls of the element and also the arrangement of the walls in the development changes. In Part A, the situation where the observer of a cubic element looks at a cube in which the front wall is the front view was clearly easier—tasks 1A, 3A, 5A, 7A. Similarly, in Part B, tasks B9, B11, B13, B15 turned out to be easier (Fig. 6).

**Figure 6:**
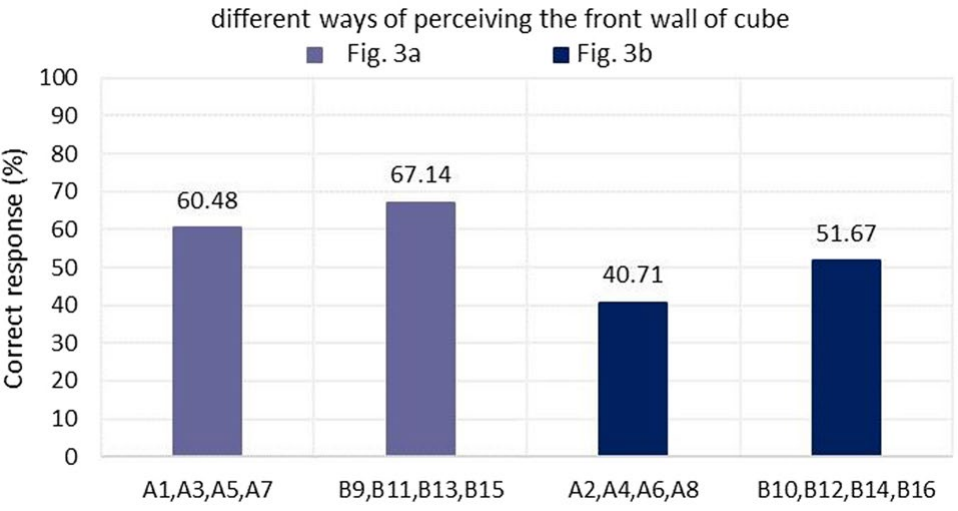
Student scores obtained depending on how you look at the cubic element and front wall view.
3. The third criterion involved a breakdown by type of high school completed (Fig. 7), with a distinction between high schools (HS) and technical schools (TS), and also by student age (Fig. 8):
  in section AI—55.64% correct answers for TS vs. 54.05% for HS;
  in section AII—46.77% correct answers for TS vs. 46.62% for HS;
  in section BI—62.90% correct answers for TS vs. 54.72% for HS;
  in section BII—70.16% correct answers for TS vs. 58.11% for HS.

**Figure 7:**
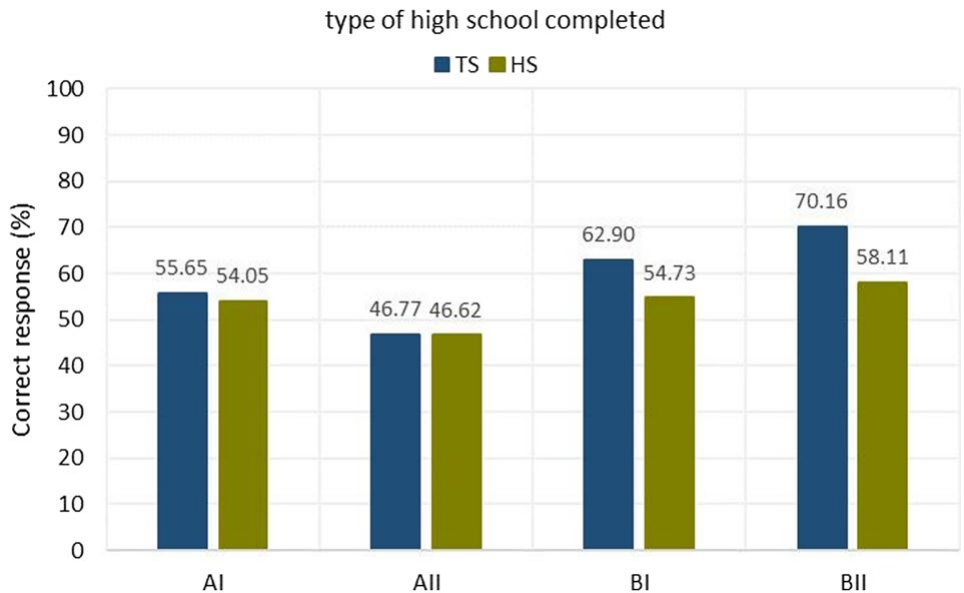
Graph with percentage of correct answers by division to a finished high school (HS) and technical school (TS).

**Figure 8:**
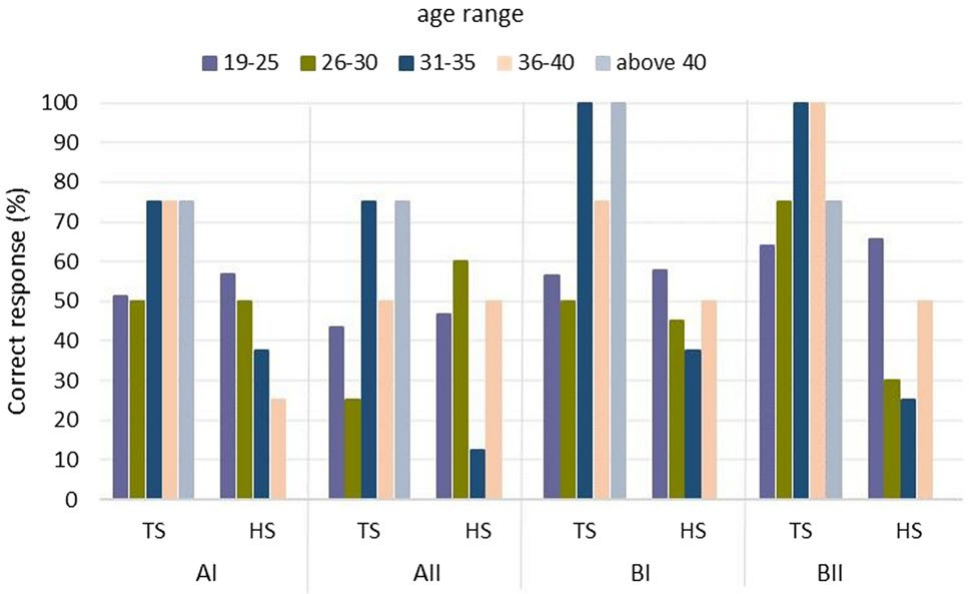
Student scores obtained—percentage of correct answers by division to a finished school and age.
  In each of sections AI, AII, BI, and BII, those who graduated from secondary technical schools generated more correct answers of those who graduated from high schools. In order to obtain data on the results obtained according to age and type of school completed, Fig. 8 was drawn up. It shows that the best performing group of students who graduated from technical schools is in the age range of 31–35 years. The correctness of answers in section B is 100% in their case. Students who graduated from technical school in the age range of over 36 did well. Students between the ages of 36 and 40 years old in section BII scored 100% correct answers. So did students over 40 years old in section BI.
4. The fourth criterion is the evaluation of the solutions of sections AI, AII, BI, and BII by gender (Fig. 9)

**Figure 9:**
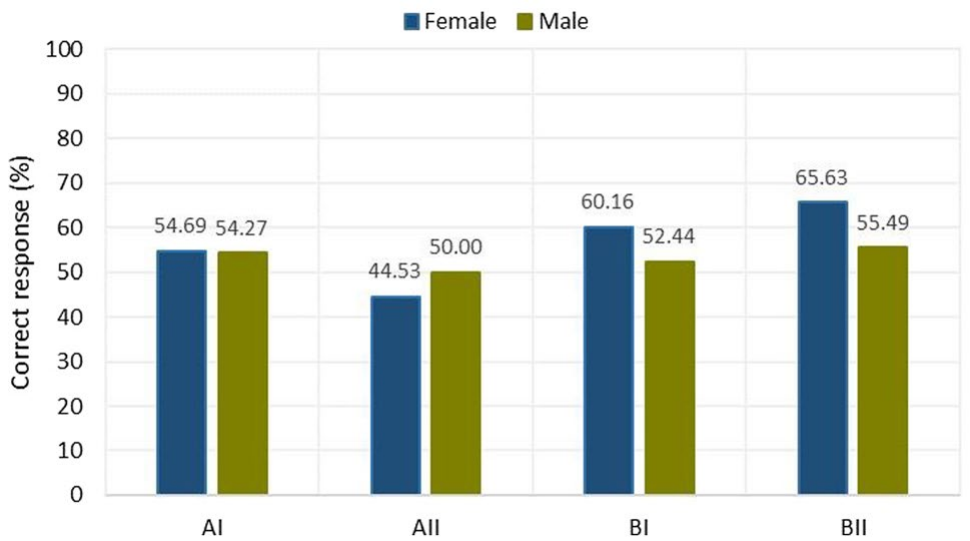
Results on correct answers given according to gender division of participants.
  The average of correct answers was significantly better for women in section B and AI, in which they obtained:
  in section BI—60.16% of correct answers by women against 52.44% by men;
  in section BII—65.62% to 55.48%, respectively;
  in section AI—54.68% to 54.27%, respectively.
  In section AII, results were 50.00% correct answers by men and 44.53% correct answers by women.
  Overall, summing up parts A and B of the test due to the gender division of the subjects and the type of tasks used:
  part A of the test yielded results—women 49.61% correct answers and men 52.13% correct answers;
  part B of the test yielded results—women 62.89% correct answers and men 53.96% correct answers.
  In the case of the breakdown of easy questions for the AI and BI sections, the results were as follows:
  women got 57.42% correct answers while men got 53.35% correct answers.
  For the breakdown of difficult questions for sections AII and BII, the results were as follows:
  men obtained 52.74% of correct answers while women obtained 55.08% of correct answers.
5. The criterion refers to the breakdown by professional experience (Fig. 10). The percentage of those who have project experience was 24.76%. Those who are working received a higher number of correct answers (53.85% in Part A), compared to those without professional training (49.52%). Similarly, in Part B, more correct answers (59.61%) were given by working students. Students without professional preparation obtained slightly fewer correct answers (59.33%).

**Figure 10:**
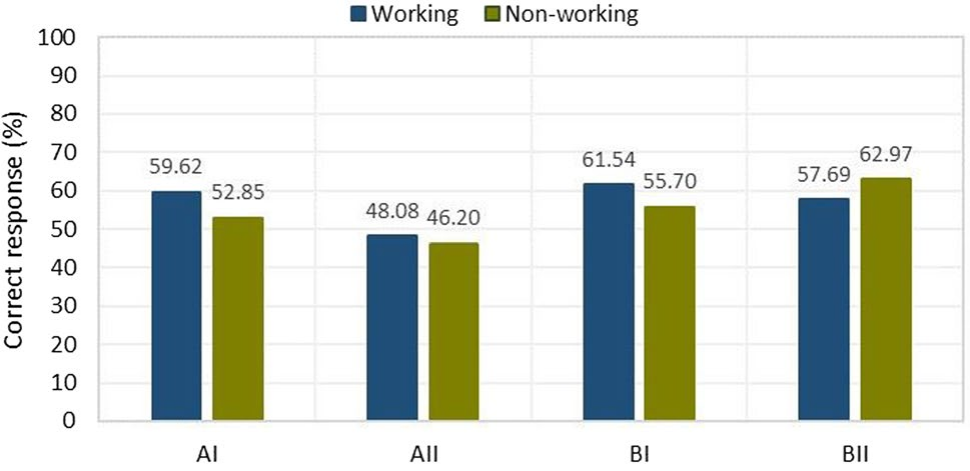
Results on the percentage of correct answers given by working and non-working people.

### Validation of the test—statistical evaluation

- Research question (RQ2) Is there a difference between students graduating from high schools and technical schools in terms of the test results obtained?
  Research hypothesis—students graduating from technical schools achieve, on average, better results on the test compared to students graduating from high schools.

The analyzed variables (total points) were subjected to the Shapiro–Wilk (SW) test (Table 3). For both groups technical schools (TS) and high schools (HS), the distribution of results differs from the normal distribution (p SW for TS is equal to 0.0035, p SW for HS is equal to 0.0044), which provides grounds for rejecting the null hypothesis about the normality of the distributions of the analyzed variables.

**Table 3:** Results the Shapiro–Wilk test (variable—total points) for the normality of distribution.

Descriptive statistics	N valid	Average	Mediana	Min	Max	Standard deviation	p
(TS)	31	9.4193	9.0000	5.0000	14.0000	2.8327	0.0035
(HS)	74	8.5405	9.0000	1.0000	13.0000	2.9011	0.0044
Female	64	9.3441	9.0000	3.0000	14.0000	2.8619	0.0281
Male	41	7.7878	8.0000	1.0000	13.0000	3.1632	0.0143
Working	26	9.2769	9.0000	3.0000	14.0000	3.1103	0.0053
Non-working	79	8.2889	9.0000	1.0000	14.0000	2.9489	0.0096

Therefore, in order to enable testing to check whether the observed differences between groups are statistically significant, a Mann–Whitney U-test (MWU) was performed. The results of the analysis are presented in Table 4.

**Table 4:** Results the Mann–Whitney U-test (variable—total points).

Relative to the variable	N valid		Sum. of ranks		U	Z	p	Z correct	p
(TS)	31		1959.0000		831.0000	2.2199	0.0264	2.2417	0.0250
(HS)		74		3606.0000					
Female	64		3706.5000		997.5000	2.0657	0.0388	2.0851	0.0371
Male		41		1858.5000					
Working	26		1646.5000		758.5000	1.9896	0.0466	2.0089	0.0445
Non-working		79		3918.5000					

Marked results are significant with p < 0.0500. Value of statistics (U for small sample size < 20; Z for large sample size > 20).

The analysis of the results presented in Table 4 provides grounds for the conclusion that there is a statistically significant difference between the test results obtained by students graduating from technical schools and those graduating from high schools. Based on the analysis carried out, it can be concluded that there is a difference between students graduating from high schools and technical schools in terms of the test results obtained in favor of students graduating from technical schools.

- Research question (RQ3) Is there a difference in the test results due to a gender?
  Research hypothesis—Women achieve better test results than men.

The analyzed variables (total points) were tested (SW), for both sexes the distribution of results differs from the normal distribution (p SW for women is equal to 0.0281, p SW for men is equal to 0.0143), which provides grounds for rejecting the null hypothesis about the normality of the distributions of the analyzed variables. The results of the analysis are presented in Table 3.

Therefore, in order to enable testing to check whether the observed differences between groups are statistically significant, a test (MWU) was performed. The results of the analysis are presented in Table 4. The analysis of the results presented in Table 4 provides grounds for the conclusion that there is a statistically significant difference between the test results obtained by women and men. Based on the analysis performed, it can be concluded that there is a difference between genders in terms of the test results in favor of women.

- Research question (RQ4) Is there a difference in the test results between students with and without work experience?
  Research hypothesis—People with professional experience achieve better test results compared to people without such an experience.

The analyzed variables (total points) were tested (SW), for both cases the distribution of results differs from the normal distribution (p SW for people with professional experience is equal to 0.0053, p SW for people without such an experience is equal to 0.0096), which provides grounds for the rejection of the null hypothesis about the normality of the distributions of the analyzed variables. The results of the analysis are presented in Table 3.

Therefore, in order to enable testing to check whether the observed differences between groups are statistically significant, a test (MWU) was performed. The results of the analysis are presented in Table 4. The analysis of the results presented in Table 4 provides grounds for the conclusion that there is a statistically significant difference between the test results obtained by people with and without professional experience. Based on the analysis carried out, it can be concluded that there is a difference between the given groups and the test results in favor of people with professional experience.

## Discussion

The way the test is presented—spatial model and its development (flat net) presents the correlation occurring in engineering projects between the model and the drawing (object realization and design) and their interrelation. The conduct of the test was very important for conducting further work with STEM students. This provided an answer to the second research goal (RG2) presented in the paper as well as the research question (RQ1) formulated in the introduction. The results were important because they showed (Figs. from 5 to 10) that many students in the study group had problems with spatial representation. This finding is particularly evident in Fig. 5, overall the students produced 55% correct answers from all tasks on average. Based on this result, several solutions were proposed to students during the second semester. The implementation of these solutions resulted in improved teaching efficiency.

The first is to strengthen the emphasis on the ability to work and use a spatial model, to analyze it. Making spatial models for the tasks completed in class, represented on a flat sheet of paper is very constructive. It helps the student understand how a geometric figure looks in space, for example.

The second solution was holding a follow-up workshop (consultation) for those who received the lowest test scores. During the workshop, there was opportunity to repeatedly correct errors and work step by step while consulting with the instructor at any time. Such a system allowed the elimination of mistakes made with simultaneous clarification, which increases the likelihood of raising the level of awareness and gives better results in further work.

The third solution was the introduction of a greater emphasis on teamwork, which provided an opportunity for students to adjust their abilities and knowledge to the level of others, while consulting the teacher. An undoubted advantage of this method was the opportunity for students to improve their own competence through group competition. Working in small groups forces active behavior and practical activities.

The obtained results of the study have also become a reason to open discussion on the introduction of additional tasks of higher difficulty. Students who wish to voluntarily expand their knowledge beyond the scope of the topics addressed in the course will have this opportunity. This is important, for example, for students who are doing well in their subject classes or have received high test scores. This allows them to get more training when the level of their lab group isn't high. Experience in teaching has repeatedly indicated that the level of laboratory groups can vary greatly. The test makes it possible to select people who may be eager for such a form of knowledge acquisition.

The test presented in this paper helps to quickly identify students who do not have a well-developed spatial imagination when starting their studies. The additional activities we have prepared (consultations, workshops, working with a physical model) help to even the chances for students. The subject of descriptive geometry and engineering graphics is one of the more difficult courses. Students often struggle with the change of environment and people and on top of that they have to quickly learn issues related to spatial abilities. It happens that the difficulties encountered with this subject are the reason for reluctance and discontinuation of studying.

The contribution to the literature is very important from our point of view, as we want to share the conclusions of our research and also the test (Appendix 1). The didactic community can use it for teaching purposes and perhaps it will also facilitate the work of other colleagues from other institutions in the world. In this paper, we share our research on the courses taught at the Faculty of Architecture; however, the authors have experience in teaching descriptive geometry and engineering graphics at other faculties such as Civil Engineering, Mining and Environmental Engineering. The conclusions from teaching students are very similar in each of these faculties. Therefore, the presented material can be a valuable source of information and the universal test can be an important didactic tool for other researchers teaching the courses of descriptive geometry and engineering graphics.

## Conclusions

The test presented is an extension of those currently in use (MRT; DAT:SR; Dimensional Cube 3DC; PSVT-D; PSVT-R; PSVT-V). The test (Appendix 1) includes elements concerning the rotation of a spatial element, the analysis of individual views, and their development on a flat sheet of paper. The main result of the study and at the same time the fulfilment of the set research goal (RG1) is a tool for both academics and students, which allows them to test spatial ability in terms of spatial perception and correctness of practical thinking. This is closely related to the acquisition of skills of the rotation of a spatial element in thought, the correct determination of views, and the creation of expansions of a spatial element. The test received very positive feedback from students.

The test results informed further teaching and educational activities for the studied group of students. Information was obtained on how to direct the conduct of further instruction in the subject of drafting geometry in architectural design and prepare the students for further work.

In addition, knowledge was obtained about the differences among students in visual perception according to age, gender, and design experience thus fulfilling the third research objective (RG3). The interpretation of the results and the main conclusions are as follows.

- Task 9 was the easiest, while Task 6 was the most difficult (Fig. 5). Section AII was the most difficult, with only 46.66% correct answers, while section BII was the easiest, with 61.66% correct answers. For section A, students gave 50.59% correct answers compared to 59.40% correct answers for section B. Based on this result, it can be concluded that the transition from flat drawing to axonometric drawing was easier for the surveyed group of students. It was surprising that none of the participants received the maximum number of points, but no one received zero points either.
- Considering the way of observing the cube, the situation where the observer of the cubic element looks at the cube, in which the front wall is the front view, turned out to be easier (Fig. 6), 63.80% of correct answers were obtained.
- The results obtained in terms of school completed clearly indicate that in the entire test, students after technical school received better results (Fig. 7), which confirms the fact that technical schools better prepare students to study engineering. However, the results in the AI section were only slightly better. This is likely influenced by the fact that working remotely during the pandemic lowered the level of student in technical schools, where classes in technical subjects were taught in a limited online format. The worst score in the entire test was achieved by students in section AII in the 31–35 age group after graduating from high school with a score of 12.50%. The result is surprising, since the same age group after graduating from technical school did very well in the entire test.
- For men and women, the questions where the line drawing is located on 3 walls are easier (Fig. 9). In summary, the difference between the results obtained was insignificant, however, overall, women did better with the tasks in both part A and part B.
- Working students did better overall in solving the prepared test (Fig. 10), receiving a score of 56.73% and non-working students 54.43%. The study also shows that depending on the difficulty of the questions for the easy questions, the AI and BI sections were better answered by working students with 60.58% correct answers. Students who are not working received a score of 54.27%. For the sections with more difficult questions AII and BII surprisingly working students fared worse received a score of 52.88% compared to non-working students who gave correct answers of 54.59%.

Based on the statistical analyses, answers to the research questions and research hypotheses were obtained (Table 4). The all research of hypothesis was confirmed:

- (RQ2) there is a difference between students graduating from high school or technical school and the test result obtained in favor of students graduating from technical school.
- (RQ3) there is a difference between gender and the test result in favor of women.
- (RQ4) there is a difference between the given groups (working and non-working) and the test result obtained in favor of people with professional experience.

The conduct of the test and the conclusions obtained had a significant impact on the way the teacher conducted semester II—second research objective (RG2). The focus pivoted more to tasks in which students analyzed the transition from a spatial element to a drawing on a plane. In addition, tasks were introduced in which it was necessary to work with the rotation of a spatial element and the mental analysis of its views. These tasks were dedicated mainly to those who received poor results in tasks A2, A4, A6, A8, B10, B12, B14, B16. Those with technical school or work experience who scored well in the test were encouraged to work in teams with students after high school. Students with the lowest scores were offered to participate in consultation classes and work with a physical model as described in the discussion section.

The test itself, as well as the results obtained, can be a valuable source of knowledge about how to teach a student in subjects related to applications of descriptive geometry and engineering graphics. Teachers can quickly get information about the level of the group with which they will work throughout the semester. This gives an opportunity to adjust assignments according to the group in question, as well as to be a teacher who understands early in the course the needs of the group. Beginning STEM teachers not infrequently need effective support during the instructional process^59^; a test can help.

For the general student population, on the other hand, the test is a good way to assess their knowledge and find areas for improvement. It also encourages the use of consultations in the early stages of the course. The testing tool supports the processes of learning, teaching, and development of students and teachers.

Further work should focus on completing a pre- and post-test for the second semester of the engineering course. The test at the end will have different tasks, but presented in the same way as in the first test. This procedure will provide quantifiable data on how much the entire course has improved spatial ability.

Another direction of research, which will be presented in a future paper, is the preparation of a test for STEM students STEM which will be checked by the students themselves. This self-test will allow them to independently analyze and evaluate mistakes based on a physical model which can be used to find the correct answers. This approach teaches students to work analytically and to identify problems with which they have difficulty. To further improve the test, the graphic form of the questions should be refined to improve the readability of the individual cases.

The overall assessment on confirms that spatial imagination is an individual property of each person. The work on this subject resulted in the implementation of the novel net cube imagination test, from which answers were obtained on the level of students' spatial perception after the first semester of the course was completed. The results informed further instruction in the subject of drafting geometry and engineering graphics for the surveyed group of students (semester II).

The modifications made in the teaching of the course had a good effect in the overall evaluation for the whole course. Students (participants) received final grades:

- 26.53% grade A;
- 12.24% grade A−;
- 38.77% grade B;
- 12.24% grade B−;
- and 10.20% grade C.

Average grade is B, which can be read as a good indicator to continue this testing practice in the following years. The students themselves commented positively on the form of the test and the subsequent conduct of the course. The test is a useful tool for academics to study spatial abilities and improve teaching activities in subjects related to geometry and engineering graphics. In addition, it serves to improve STEM education.

### Supplementary Information

Supplementary Information.

## Data Availability

The dataset generated and analyzed for the current study is not publicly available due to privacy reasons but is available from the author on reasonable request.
