# Supplementary material for: Evaluation of STEM students' spatial abilities based on a novel net cube imagination test
Source: Sci Rep. 2023 Oct 12;13:17296. doi: 10.1038/s41598-023-44371-5 (PMC10570322; doi:10.1038/s41598-023-44371-5)
Supplement: Supplementary file 1 — Supplementary Information. [file 41598_2023_44371_MOESM1_ESM.pdf]

NET CUBE IMAGINATION TEST (NC)

Gender	Female	Male
Age		
High school graduate		
Work / practice<br>in the profession	yes	no

Section A

Indicate on the basis of the spatial element and the entered cut line the correct solution of development (a flat net). The cut line determines how to observe the element and determines the front wall of the spatial element. The front wall is always located as the first on the left side in the net. Stages of unfolding a spatial element into the form of a flat net is illustrated in the examples below.

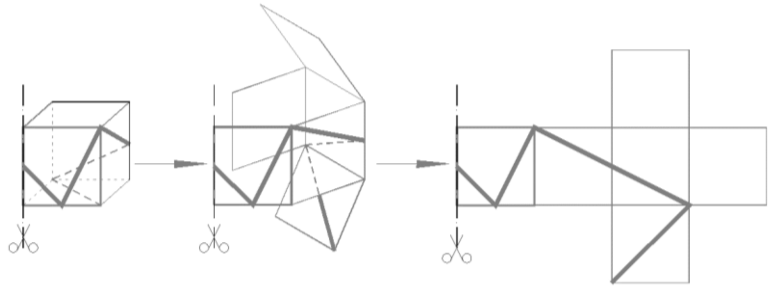

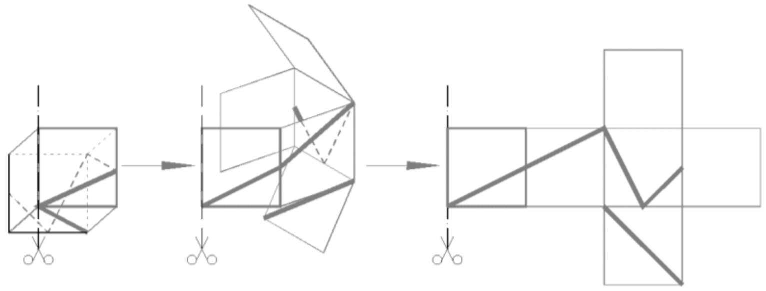

Section B

Indicate, based on the net and the introduced cut line, the correct solution for the spatial element. The cut line determines the front wall for the spatial element. The examples below is illustrated the stages of net folding.

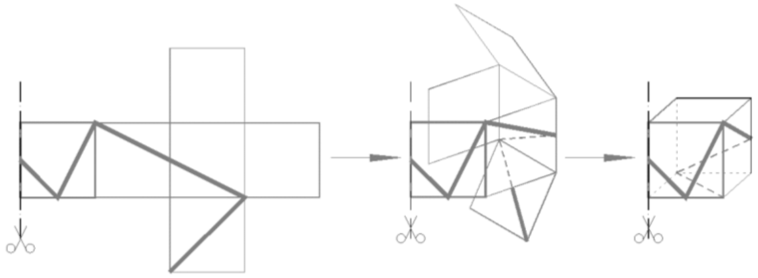

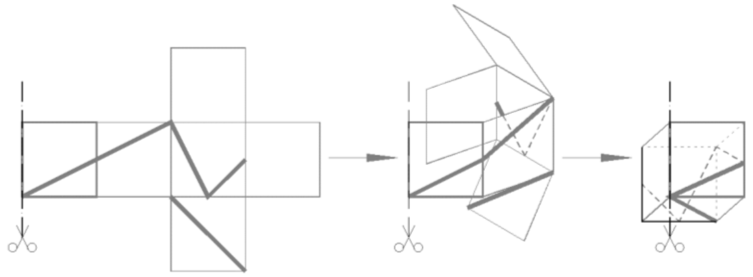

1 A	M

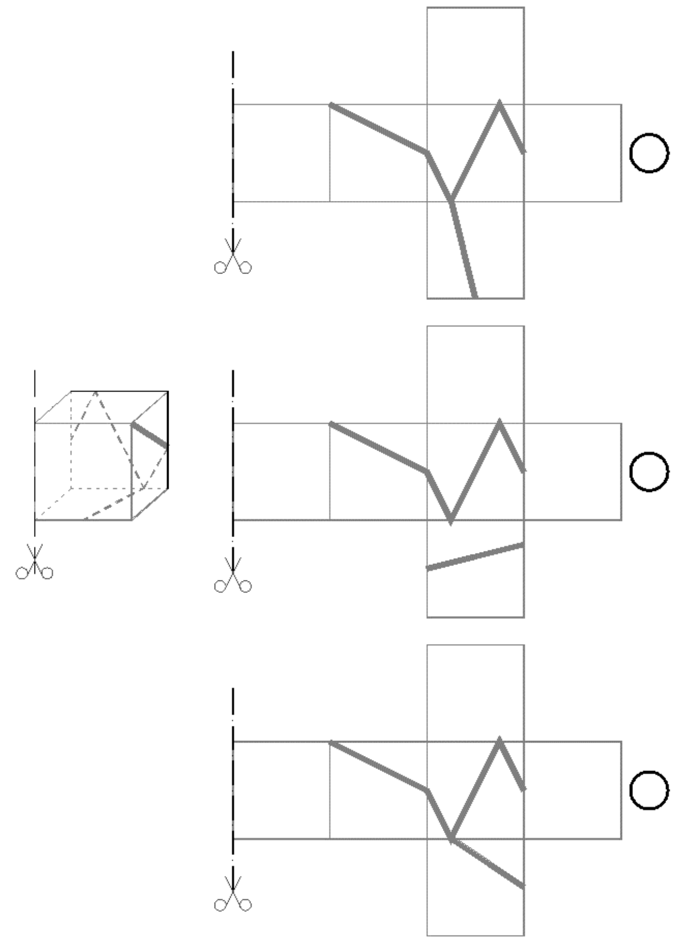

2 A	M

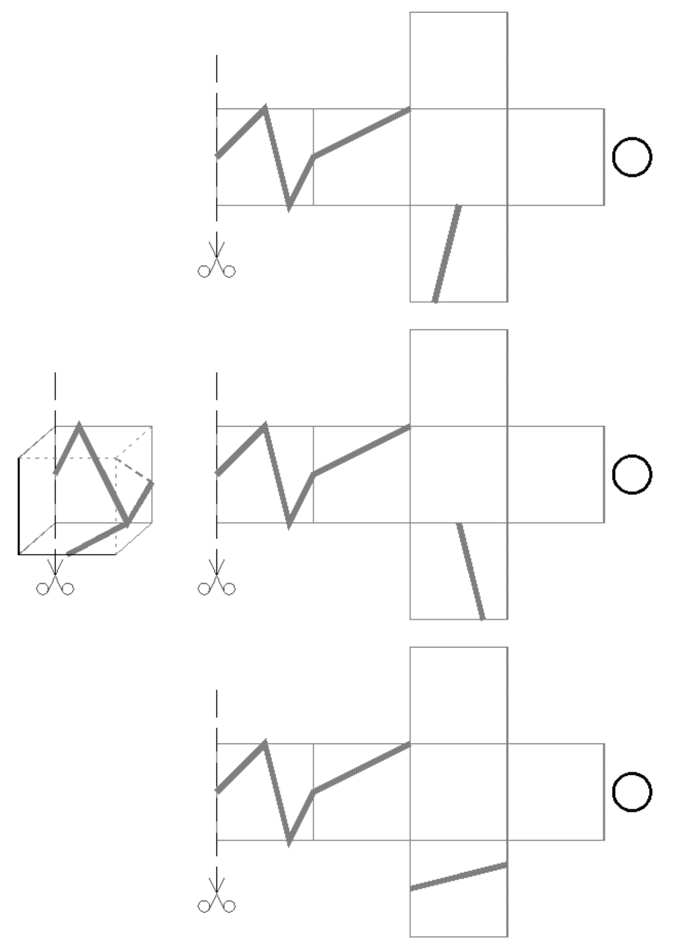

3 A	M

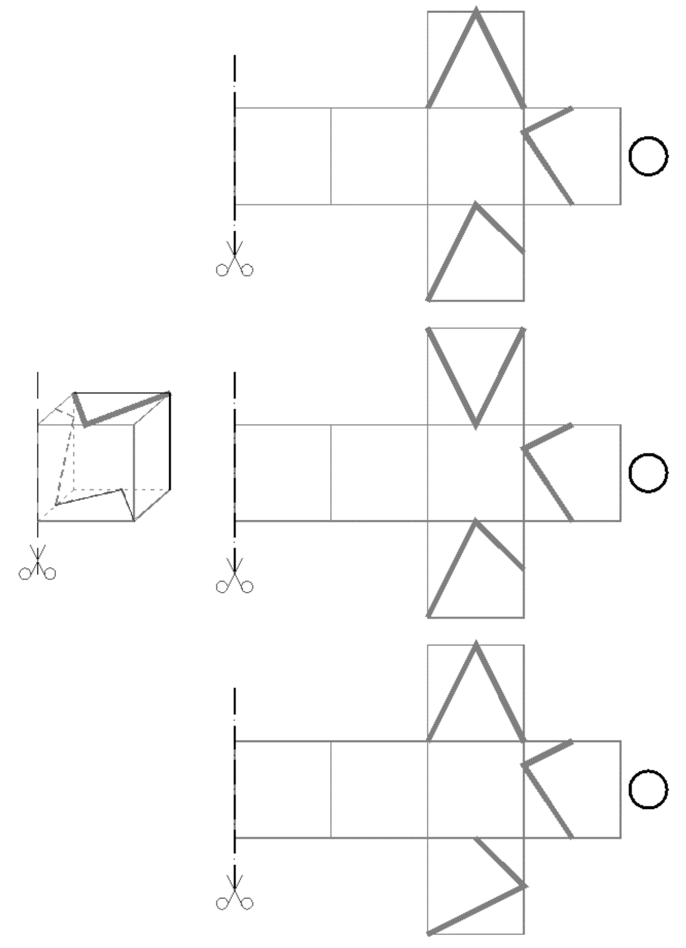

4 A	M

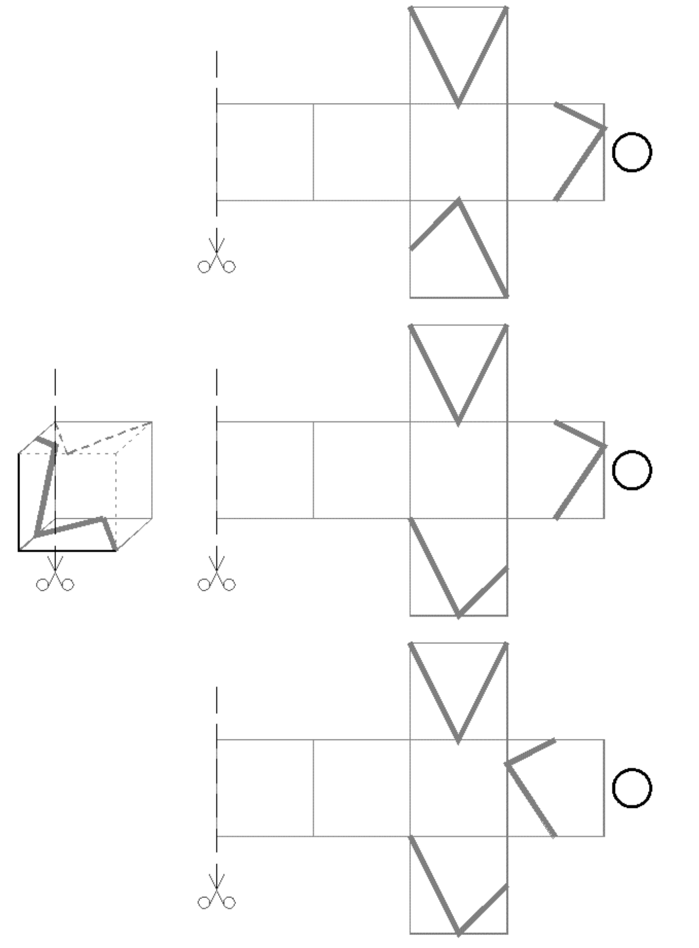

5 A	M

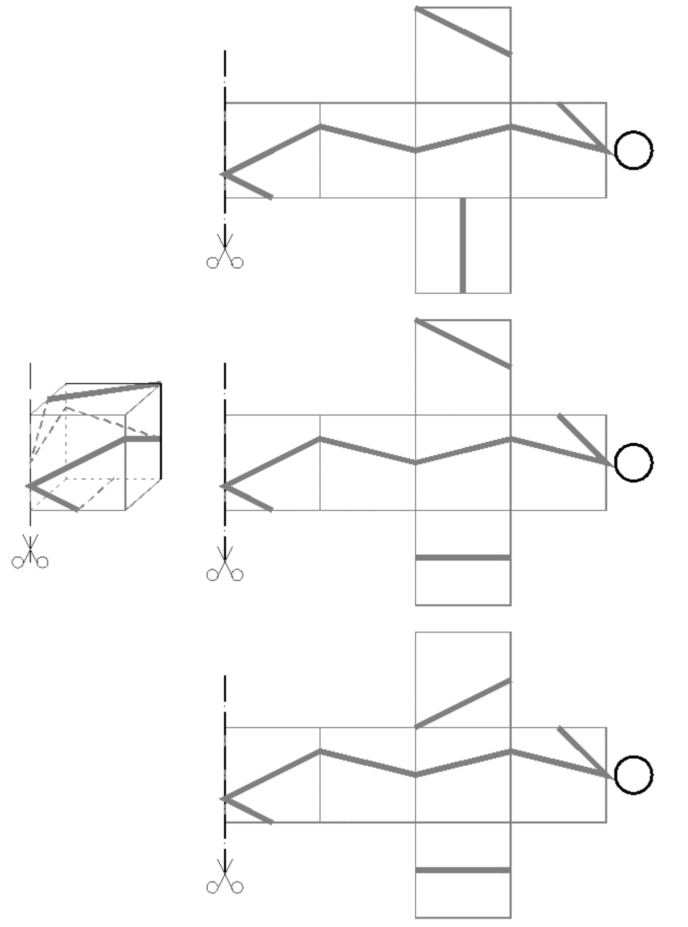

6 A	M

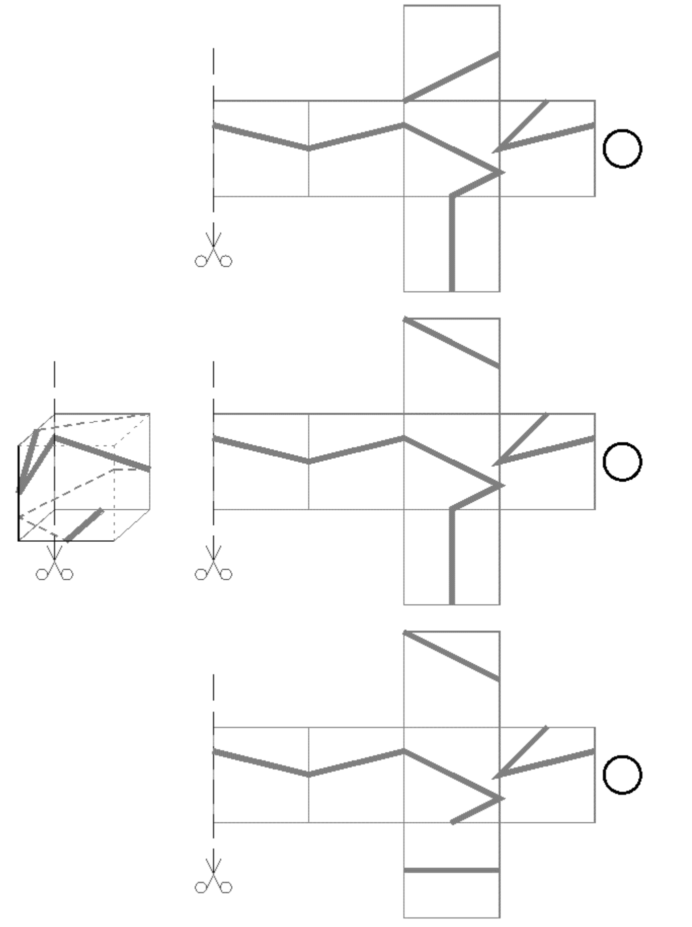

7 A	M

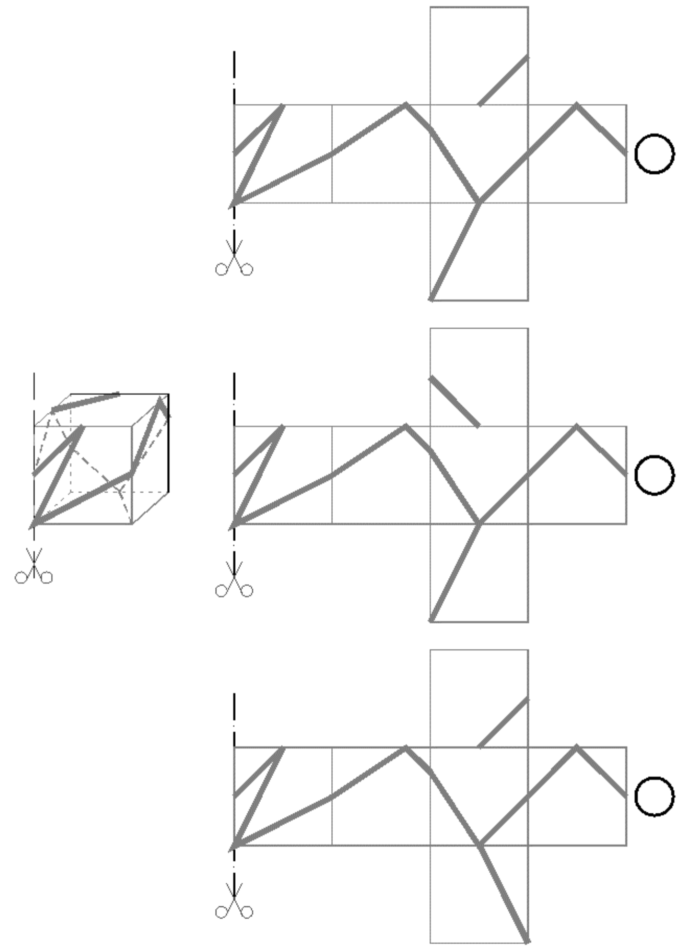

8 A	M

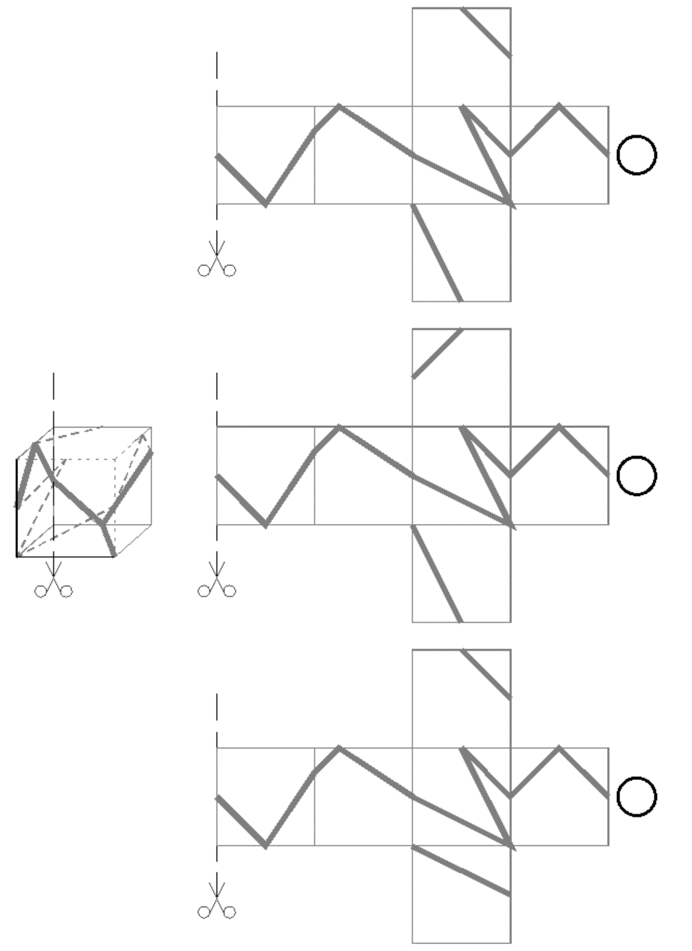

**9 B**	**M**	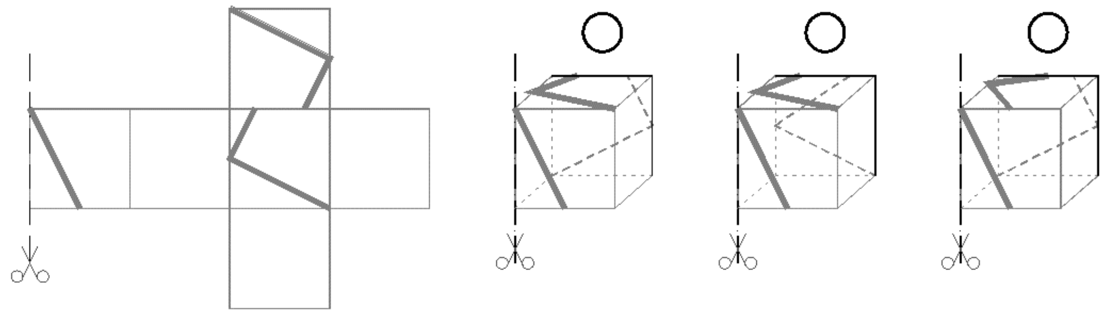
**10 B**	**M**	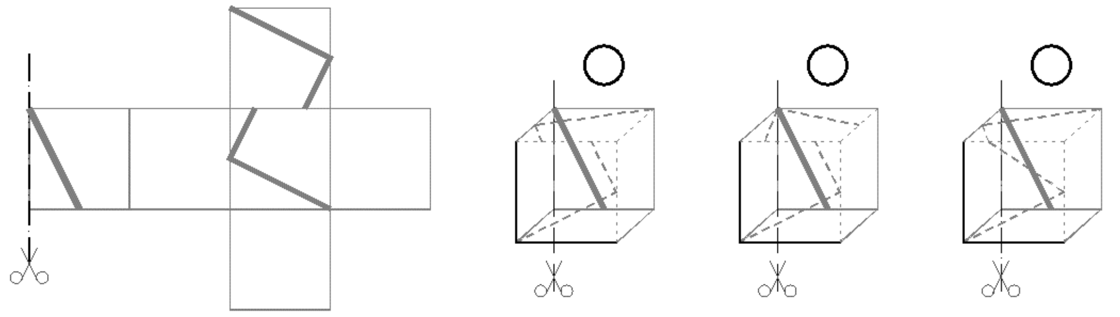

**11 B**	**M**	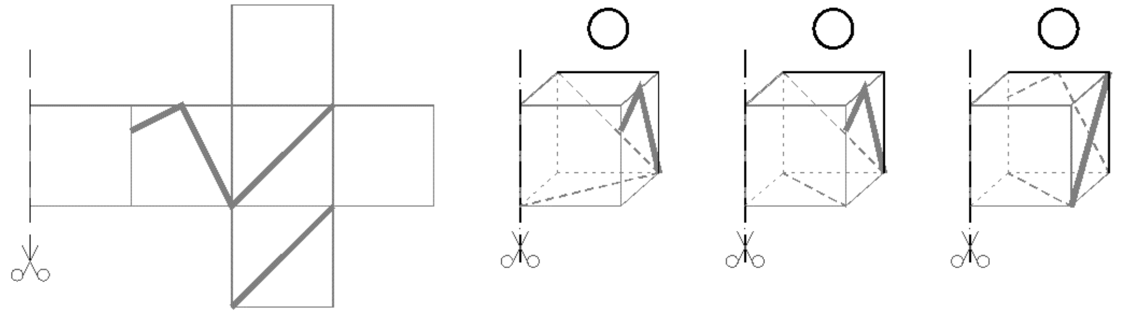
**12 B**	**M**	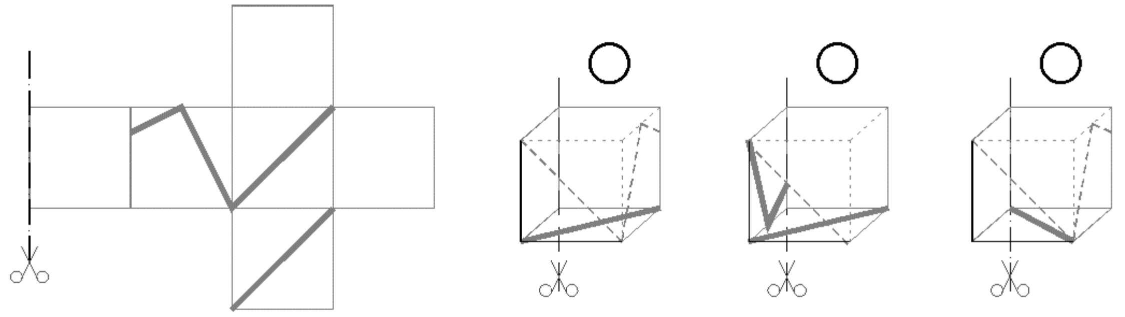

**13 B**	**M**	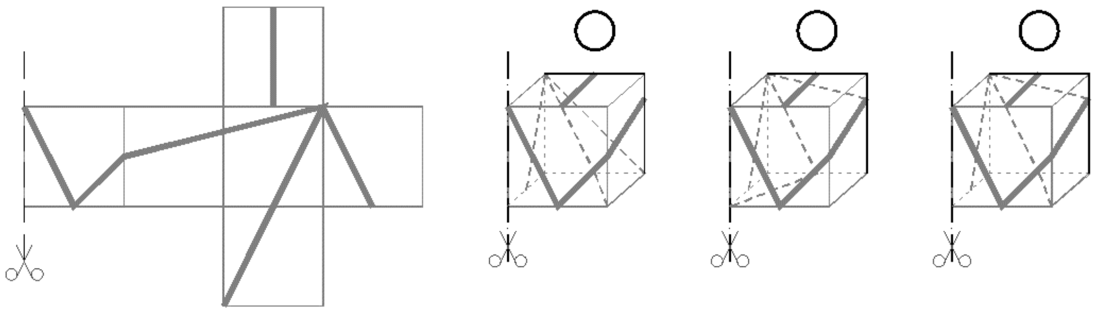
**14 B**	**M**	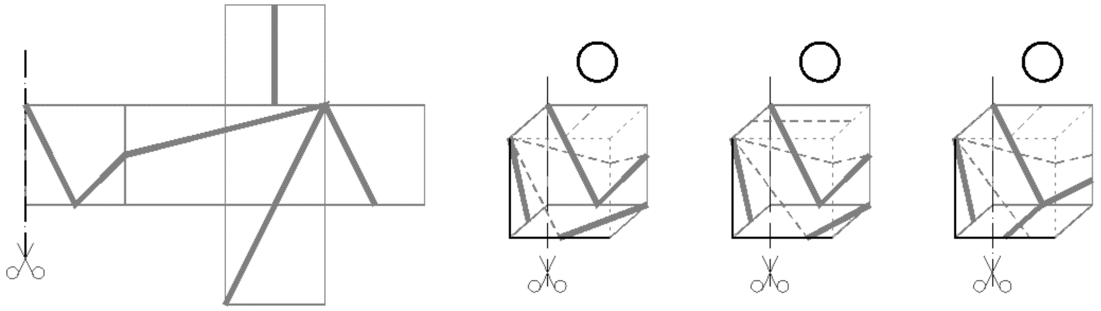

**15 B**	**M**	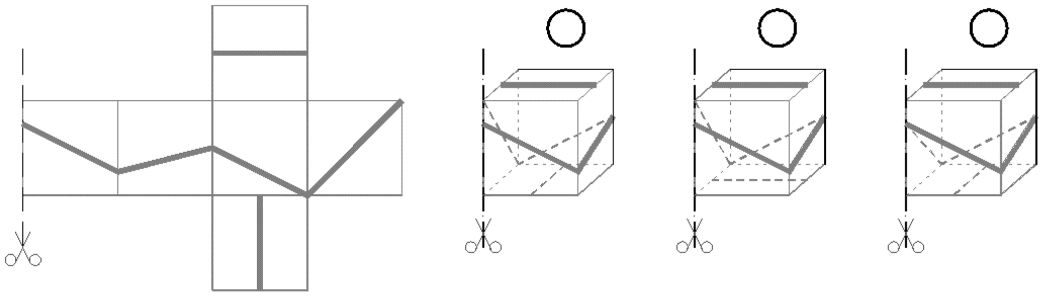
**16 B**	**M**	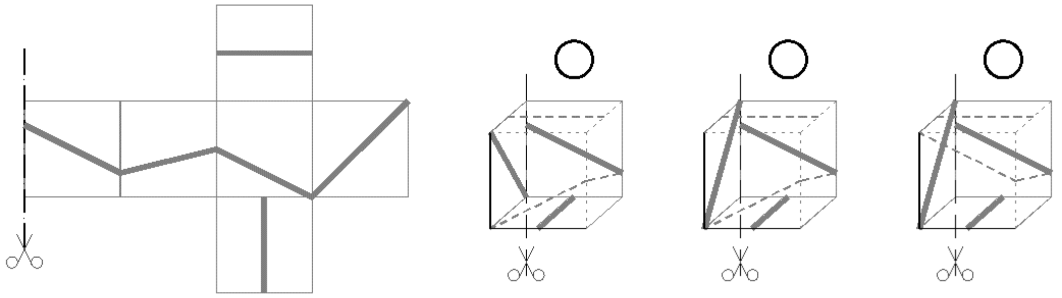
